# Supplementary material for: Pregnancies and Gynecological Follow-Up after Solid Organ Transplantation: Experience of a Decade
Source: J Clin Med. 2022 Aug 16;11(16):4792. doi: 10.3390/jcm11164792 (PMC9409658; doi:10.3390/jcm11164792)
Supplement: Supplementary file 1 [file jcm-11-04792-s001.zip › Table S1.pdf]

Table S1. Demographic characteristics of patients according to pregnancy status post-transplantation

	**Patients with ≥1 pregnancy (n=24)**	**No pregnancy patients (n=186)**	**p value**
Age at SOT, years	29.3[1] ± 5	29.2 ± 6.1	0.939
BMI at SOT, kg/m^2	19.8 ± 5.7 (*n=17*)	20.2 ± 4.5 (*n=168*)	0.734
Organ transplanted			
Kidney	15[2] (62.5%)	81 (43.5%)	0.079
Lung	9 (37.5%)	105 (56.5%)	
History of			
Hypertension	12 (50.0%)	59 (31.7%)	0.074
Diabetes mellitus	4 (16.7%)	70 (37.6%)	0.067
Gyn. cancer	1 (4.2%)	1 (0.5%)	0.216
Gravidity (G)			
G0	17 (70.8%)	116 (62.4%)	0.417
≥ G1	7 (29.2%)	70 (37.6%)	
Parity (P)			
P0	17 (70.8%)	130 (69.9%)	0.925
≥ P1	7 (29.2%)	56 (30.1%)	
History of preeclampsia	*n=4*	*n=25*	
(of n: ≥ P1)	2 (50.0%)	10 (40.0%)	1.000

[1] Values are mean ± SD

[2] Values in parentheses are the percentages of the number of patients in each transplantation group unless otherwise indicated.

BMI: body mass index, Gyn.: gynecologic.
