# Supplementary material for: Pregnancies and Gynecological Follow-Up after Solid Organ Transplantation: Experience of a Decade
Source: J Clin Med. 2022 Aug 16;11(16):4792. doi: 10.3390/jcm11164792 (PMC9409658; doi:10.3390/jcm11164792)
Supplement: Supplementary file 1 [file jcm-11-04792-s001.zip › Table S2.pdf]

Table S2. Medical characteristics of patients according to pregnancy status post-transplantation

	**Patients with ≥1 pregnancy (n=24)**	**No pregnancy patients (n=186)**	**p value**
Etiology			
**Lung**:	*n=9*	*n=105*	
CF	9[1] (100%)	95 (90.5%)	1.000
PCD	0	1 (0.9%)	1.000
Bronch.	0	4 (3.8%)	1.000
Fibrosis	0	3 (2.9%)	1.000
Other[2]	0	2 (1.9%)	1.000
**Kidney:**	*n=15*	*n=81*	
Diabetes	0	13 (16.0%)	0.210
Glom.	5 (33.3%)	20 (24.7%)	0.527
Vasc.	1 (6.7%)	4 (4.9%)	0.580
PKD	0	5 (6.2%)	1.000
Interst.	2 (13.3%)	4 (4.9%)	0.235
Unknown	3 (20.0%)	16 (19.8%)	1.000
Other[3]	4 (26.7%)	19 (23.5%)	0.751
Multi-SOT	0	18 (9.7%)	0.235
GFR[4] (ml/min/1.73m^2)	*n = 15*	*n=81*	
1 year after SOT	68[5] ± 22 (*n=10*)	59 ± 24 (*n=73*)	0.353
2 years after SOT	69 ± 16 (*n=10*)	56 ± 24 (*n=62*)	0.199
FEV1[6] (%)	*n = 9*	*n=105*	
1 year after SOT	87.6 ± 19.2	80.6 ± 17.5	0.256
2 years after SOT	86.7 ± 21.3	82 ± 18 (*n=103*)	0.458

[1] Values in parentheses are the percentages of the number of patients in each transplantation group unless otherwise indicated.

[2] Includes emphysema, bronchiolitis

[3] Includes urological malformation, side effects of use of calcineurin inhibitors, systemic lupus erythematosus, scleroderma, Alagille syndrome, acute tubular necrosis, tuberous sclerosis complex, hemolytic uremic syndroma, bilateral persistant obstruction, hyperoxaluria

[4] Normal GFR is ≥ 90 ml/min/1.73m^2 for a young woman, eGFR : estimated according to CKD EPI equation.

[5] Values are mean ± SD

[6] Lower limit of normal FEV1 is 80% in the general population. Pregnancies were usually prohibited for women if the FEV1 was under 40%.

CF: cystic fibrosis, PCD: primary ciliary dyskinesia, bronch.: bronchiectasis, glom.: glomerulopathy, vasc.: microvascular condition for hypertension, PKD: polycystic kidney disease, interst.: interstitial kidney disease
